# Mentoring Nurse Scientists to Meet Nursing Faculty Workforce Needs

**DOI:** 10.1100/2012/345085

**Published:** 2012-02-14

**Authors:** Mary A. Nies, Meredith Troutman-Jordan

**Affiliations:** School of Nursing, College of Health and Human Services, University of North Carolina at Charlotte, 9201 University City Boulevard, CHHS 416, Charlotte, NC 28223, USA

## Abstract

Research indicates that mentoring has been highly effective in promoting faculty success. Strong mentors in the area of scholarship are extremely valuable for junior faculty, not only because of their research and academic expertise but also for their role modeling behaviors. This paper highlights key components of research mentoring used by a senior nursing faculty member. The senior faculty mentor and junior faculty mentee developed a common vision, relating to research interests in health promotion for vulnerable populations. Impact at the individual, school, university, and society level is discussed, and benefits of mentoring to meet nursing faculty workforce needs are emphasized.

## 1. Introduction

In light of the shortage of nursing faculty, it is imperative that current faculty be retained. An essential part of retaining faculty involves fostering the professional growth of junior faculty members. The presence of an endowed chair is particularly vital to the success of junior faculty members: an endowed chair can provide research opportunities to gain high visibility for the nursing discipline, advance a focused research program, and create an environment for nurturing novice faculty members [1]. Quality mentoring is one mechanism by which this can occur [2–4]. This paper discusses the role of an endowed chair in mentoring a junior faculty and the mutual benefits of the mentoring process.

 Fitzpatrick and Carnegie [5] describe critical elements of the mentor role, which include inspiring young academicians and promoting freedom and opportunities for nursing scholarship, research, and the profession. As mentors, endowed chairs are enabled to affect the nursing, scientific, and lay communities [1]. A good mentor can assist the mentee in multiple ways: providing advice on a program of scholarly development, offering opportunities for this development, and engaging other scholars in mentoring and collaborative activities [6].

## 2. What is Mentoring?

Mentoring involves a relationship between two people in which one person with greater experience and/or expertise teaches and counsels the other to develop professionally [7]. The relationship lasts over an extended period of time and is distinguished by commitment of both parties [7]. The mentoring relationship is a voluntary alliance for the dual purpose of career development and the enhancement of the profession [8]. It includes supportive and nurturing elements [9].

 Typically one would expect a more senior nurse scientist in the role of mentor and a junior nursing faculty member as mentee, though peer-to peer mentoring is becoming increasingly common as the result of faculty shortages, increasing workloads and budget limitations [10]. Regardless of the mentor's seniority, mentoring is beneficial to the mentor and the mentee [10]. The authors of this paper suggest that mentoring involves a “passing on” of the experience, expertise, and counsel cultivated from the mentoring relationship to more junior faculty members. This generative notion of mentoring can be one part of the solution to the nurse faculty shortage; there is greater potential for retention of current faculty members and recruitment of graduate students as new faculty members if novice nurse scientists are cognizant of and experience positive, beneficial mentoring [11, 12].

Trust is an essential ingredient for a successful mentoring relationship; in all advising settings, advisors seeking to guide and nurture others must first establish trust [13]. Covey [14] notes the importance of establishing and extending trust as the most effective way of relating to and working with others and the most effective way of getting results. Once trust is established, honest communication can occur and potential challenges such as time constraints can be discussed, facilitating a teamwork approach on the part of the mentor and mentee.

Heinrich [15] describes positive mentoring behaviors that nurse mentors can adopt to nurture mutually beneficial relationships and create an environment of respect, productivity, and self-worth. One of these behaviors is “guiding others”; this responsibility is undoubtedly the most salient for the mentoring experience. The level of guidance mentees receive can directly affect their success. However, mentees must be receptive to guidance. Heinrich also notes that mentors must reflect positively on the person being mentored. Reflecting positively [16] requires skill and careful communication, in order for feedback to be accepted as constructive and growth producing, rather than as discouraging criticism. Heinrich further identifies being transparent, being respectful, conveying expectations and norms directly, honoring boundaries; respecting diversity, seeking win-win solutions, assuming personal responsibility; and encouraging participation and inclusion as essential behaviors of positive nurse mentoring.

## 3. Elements and Examples of a Mentoring Model

As mentor and mentee, the authors recognized soon after meeting that they shared a common interest in health promotion and intervention research in community settings. The mentor's community health background and related research expertise were a good fit with the mentee's research interest in successful aging and health promotion of older adults, both in the community and assisted living facilities.

Upon their initial meeting, the mentor and mentee began to identify shared interests in nursing research and discuss the mentee's career goals. They later discussed their academic institutions expectations of mentoring, outlined goals, and strategies for the upcoming academic year and made plans for how and when to meet. The process used for the mentorship was Dunham-Taylor et al.'s [12] proposed elements of mentoring.

Dunham-Taylor et al. [12] suggest ten essential elements of mentoring, which include socialization and collaboration. Socialization is described as interactional and reciprocal, where the mentor and mentee are mutually influenced [12]. Though the mentor is a seasoned researcher with far more experience than the mentee, the mentor was newly recruited to the authors' university. Therefore, the mentor-mentee pair helped each other; the mentee helped orient the senior researcher to the university, school of nursing, and its functioning, while the mentor oriented the more junior faculty member to the grant writing and submission process for federal funding. Collaboration involves becoming connected as a team and sharpening skills at teamwork [12]. The mentor demonstrated and explained how to put together a multidisciplinary research team and negotiate and delegate related tasks to team members.

Another element of mentoring is operation orientation [12], which entails explaining written and unwritten rules, policies, and procedures. While the mentor provided explanations of navigating various grant funding mechanisms (e.g., how to set up teleconferences with NIH program officers,), the mentee oriented her to the school's MSN program capstone project requirements. Together, the two discussed opportunities for enhancing their own research projects while exposing students to the research process and providing a “real life” research experience.

Validation and expectations are also components of mentoring [12]. Validation involves providing feedback and constructive criticism to improve teaching skill and self esteem. The mentor provided peer evaluations of teaching for the mentee and critiques of grants and manuscripts, while the mentee shared information she had learned previously as well as course materials for an online course, when the mentor taught the same course. Throughout the years of mentoring she has provided, the senior nurse faculty member has role modeled and encouraged healthy expectations by explaining that busy times (e.g., grant deadlines and grades due) occur at certain times of each semester [12] and during the grant writing process.

Another important element of mentoring is transformation, encouraging professional growth through reading and attending educational conferences [12]. The mentor regularly offered guidance on professional organization conferences to attend and to apply for the NIH Office of Social Sciences and Behavior Research annual summer institute and she and the mentee share relevant research articles with each other ongoing. The mentor models participation in activities including service on school, college, university, and professional organization committees, student support (chairing dissertation committees and reviewing papers), research (inducted into Sigma Theta Tau International Nurse Researcher Hall of Fame); and teaching excellence. All of these activities have contributed to the advancement of either individual's research, through networking opportunities that resulted in connecting with coauthors, peer-reviewers, individuals to provide letters for tenure dossier, and community collaborators.

Exhibited behaviors have effects on the reputation of nursing; the mentor can discuss appropriate and inappropriate behaviors [12]. The mentor demonstrates behaviors for the academic and local community, serving as chair on various committees and discussing with the mentee her role as a grant reviewer, manuscript reviewer, and leader.

Another vital component of mentoring is documentation; faculty members need to know the importance of maintaining a method of organizing and tracking documentation necessary for licensure, promotion, and tenure. The mentor and mentee carefully planned research and service activities to prepare for the tenure review process, and how to document these. Further, the mentor worked with the mentee to develop a system for tracking manuscript plans, development, and submission status. Similarly, the mentor and mentee developed a system for documenting grant application status: from idea, to development progress, to submission. The mentee has obtained two externally funded grants while under the mentorship of the mentor.

Generation, using one's wisdom and experience to help more junior faculty, is yet another element of mentoring [12]. Regularly, the mentor shares what she has learned with the mentee. For example, the mentor counseled the mentee on allocation of time and energies for service pretenure and the need to delay service on some committees until after tenure. The mentee has moved from pretenure to obtaining tenure during the mentoring process. In turn, the mentee has been able to share her knowledge of electronic technologies useful when teaching on line and strategies for connecting and communicating with online students.

Lastly, Dunham-Taylor and colleagues [12] suggest perfection as a key element in mentoring. Perfection is an inappropriate expectation, they assert, suggesting the mentors prevent mentees from having this expectation of themselves by making sure the mentee knows that perfection is not an expectation. The mentor has shared her experiences with the mentee regarding grants that were not funded and manuscripts that were rejected, sharing that perfection should not be an expectation.

## 4. Outcomes of Mentoring

Advantages of mentoring for both the mentors and the mentees might be the academic acceleration both parties receive. The mentees learn from the mentor, and, in turn, the mentor learns by teaching the mentees.

 Faculty mentoring of students can be especially rewarding for students learning vicariously through the mentor's experiences in nursing. This gives students a direct advantage in their class work and studies. At the authors' institution, graduate level nursing students experience faculty mentoring and are socialized to the researcher role as they work on their evidence-based capstone projects. Each student is paired with a project advisor, who guides the student through the steps of a research project. As mentors, both authors explained the steps of the research process to graduate students, socializing them to the role of nurse researcher, and demonstrating activities such as how to recruit research participants and obtain informed consent, and how to conduct statistical analysis and interpret these findings. The graduate students were able to observe the mentor and mentee collaborate on a research project and experience how collaboration occurs, and they became part of a team with a common interest, as the students assisted the mentor and mentee with manuscript critiques, data collection and entry, as suggested by the literature on mentoring students in research [6, 17].

As a recipient of quality mentoring, the junior faculty author has practiced the vital skills the mentor implemented through her mentoring. The “passing on” of this quality mentoring has been productive; the mentee's former capstone advisees have had papers accepted for publication and presentation, and all have obtained advanced practice nursing positions. Riley and Fearing [10] note that mentors should provide a real-world experience for nursing students that enhances their professional roles as well as their learning. The mentor-mentee pair has worked jointly with graduate nursing students on research learning opportunities and publications. The experiences provided opportunities for mentoring of the junior faculty member, and role modeling of mentoring students by the mentor, and afforded the mentor the ability to assess the mentee's mentoring of students.

Mentors also provide career information, for example, by providing feedback on strategic education enrichment opportunities such as essential professional organizations to join and allocation of time between research, teaching, and service, in order to optimize career success. Mentees are then better equipped to practice career enhancement mentoring with students. For example, the mentee has assisted her students to refine their resumes and interview skills in order to strengthen their job applications. This type of mentoring prepares students for the workforce and gives them the tools with which to seek employment after graduation. Thus, mentoring has multiple positive outcomes, for mentors, mentees, students, academic institutions, and healthcare organizations.

Another outcome of mentoring is leadership development; according to a study by Young [18], participation in leadership and mentoring programs increases mentees' leadership skills that are noticeable by others. The current authors have also experienced leadership development. For example, with the guidance of the mentor, the mentee applied to and was accepted as a faculty leader at the university's summer leadership development program for students. This experience gave the mentee an opportunity to provide guidance to future undergraduate nursing students who will eventually become members of the workforce. The mentor assumes responsibility for ensuring that the mentee learns how to balance successfully the responsibilities of academia, including teaching, research, and service in a university. Mentoring by the mentor has also enhanced the career development of other junior faculty members and enriched their experiences as faculty members facilitating their receipt of externally funded grants, publishing in peer-reviewed and indexed journals, and meeting requirements for reappointment, tenure, and promotion.

## 5. Examples of Mentoring Impact at Multiple Levels

Guided by the mentor's own experiences as a mentee and the literature on mentoring in nursing, the authors have experienced successful mentoring relationships. There have been positive outcomes at individual, school, university, and society levels. The mentee was successful in obtaining funding within the first year of the mentoring relationship (a Sigma Theta Tau International Research grant and an American Nurses Foundation grant) and thus was able to advance her program of research. The mentee has also been able to broaden her network of collaborators, through introductions to various nurse scientists and interdisciplinary research team members at national conferences, by the mentor, who has also role-modeled networking skills during these introductions.

 More broadly, successful mentoring relationships with several senior faculty members, including the current mentor, have contributed to faculty retention in the school of nursing and the university; the junior faculty mentee has remained at this university for 8 years now. The benefits of being well mentored have also contributed to the mentee's leadership skills, enabling her to make contributions on various college and university committees and serve as a member of dissertation committees.

 Beyond the university environment, mentoring has positive outcomes for the society: as mentees become more skilled in conducting research on health promotion for vulnerable populations, these research findings will have implications for clinical practice and the health of the community. For example, their research on successful aging with southern community-dwelling older adults [19] will add to the science base needed to promote the health of vulnerable populations.

 Skilled mentoring has also helped the junior faculty member establish partnerships with various members of the lay community, including local senior centers and federally qualified health centers, for research as well as service opportunities. Mentoring has facilitated dissemination of knowledge via presentations and papers, through support and encouragement the mentor provided.

## 6. Limitations of Mentoring

Mentoring can have limitations at times depending on demands on the mentor and the experience of the mentee [20]. If the mentor has excessive leadership, service, research, and teaching responsibilities, they may have difficulty dedicating adequate time and energy to mentoring. Mentors and mentees may at times feel healthy tension when the mentee feels they have learned what they can from a mentor and the mentee wishes to move on either without a mentor or obtain another mentor. Mentors may also be at risk for burnout, and if role strain and frustration occur, the mentoring experience can be unproductive for both individuals. Prior to entering the mentoring relationship, it behooves both mentor and mentee to do a self-inventory to identify the reasons for engaging in mentoring (e.g., self-identified needs, institutional expectations, and grant program requirements) and what they hope to achieve from the relationship.

## 7. Summary

Although mentoring can be exhausting at times, seeing mentees receive grants to further their programs of research, get their manuscripts published, obtain tenure, and serve the university and the greater community is very rewarding. Mentoring is an important strategy that nursing leaders can use to facilitate the growth of our future nurse scientists. Mentoring is a way to continually pass on experience to the next generation of nurse scientists and meet nursing faculty workforce needs.
